# Vasculitis update: pathogenesis and biomarkers

**DOI:** 10.1007/s00467-017-3597-4

**Published:** 2017-08-07

**Authors:** Paul Brogan, Despina Eleftheriou

**Affiliations:** 1 Infection, Inflammation, and Immunology Section, University College London Great Ormond Street Institute of Child Health, 30 Guilford Street, London, WC1N1EH UK; 2 Arthritis Research UK Centre for Adolescent Rheumatology, University College London Great Ormond Street Institute of Child Health, 30 Guilford Street, London, WC1N1EH UK

**Keywords:** Vasculitis, Child, ANCA, Henoch–Schönlein purpura, IgA vasculitis, Monogenic vasculitis

## Abstract

Better understanding of the pathogenesis and treatment of primary systemic vasculitides (PSV) has led to the development of many potentially clinically relevant biomarkers. Genome-wide association studies have highlighted that MHC class II polymorphisms may influence the development of particular anti-neutrophil cytoplasmic antibody (ANCA) serotypes, but not the clinical phenotype of ANCA-associated vasculitis (AAV). Although ANCAs are overall poor biomarkers of disease activity, they may be useful for the prediction of flares of renal and/or pulmonary vasculitis. Moreover, patients with proteinase 3 (PR3)-AAV may respond better to rituximab than cyclophosphamide. Newer biomarkers of renal vasculitis in AAV include urinary soluble CD163, and may in the future reduce the requirement for renal biopsy. Better understanding of dysregulated neutrophil activation in AAV has led to the identification of novel biomarkers including circulating microparticles, and neutrophil extracellular traps (NETs), although their clinical utility has not yet been realised. Studies examining endothelial injury and repair responses have additionally revealed indices that may have utility as disease activity and/or prognostic biomarkers. Last, next-generation sequencing technologies are revealing monogenic forms of vasculitis, such as deficiency of adenosine deaminase type 2 (DADA2), and are profoundly influencing the approach to the diagnosis and treatment of vasculitis in the young.

## Introduction

This review summarises recent advances in our understanding of the pathophysiology of primary systemic vasculitides (PSV), and consequently, the potential role of novel biomarkers discovered in that context. The diseases considered are those where renal vasculitis is of particular concern, namely the anti-neutrophil cytoplasmic antibody (ANCA)-associated vasculitides (AAV), and IgA vasculitis (IgAV; Henoch–Schönlein purpura, HSP). A brief mention of other forms of PSV, and of treatment, will be made only in the context of more general comments in relation to pathophysiology and biomarkers. For recent reviews and guidance in relation to treatment, the reader is referred to Eleftheriou and Brogan, Ntatsaki et al. and Yates et al. [1–3].

## What is a biomarker, and what is it a biomarker of?

A biomarker is defined as any characteristic that is objectively measured and evaluated as an indicator of normal biological processes, pathogenic processes, or pharmacological responses to a therapeutic intervention [4]. An equally important and clinically relevant question regarding biomarkers is “a biomarker of what?” In the context of PSV, biomarkers may have clinical utility for different non-mutually exclusive areas including: predisposition to vasculitis; diagnosis; prediction of disease flare; disease activity; prognosis; and/or choice of therapy. With this framework in mind, particular clinical issues where biomarkers and/or new genetic tests could help include: diagnostic tests to robustly identify important clinical phenotypes of vasculitis (for epidemiological studies and/or clinical trials), e.g. identifying novel genetic diagnostic tests for monogenic vasculitides, or for distinguishing between microscopic polyangiitis (MPA) and granulomatosis with polyangiitis (GPA); prediction of disease flares and/or identification of subjects at risk of frequent flares; biomarkers of disease activity to help guide treatment duration and optimal timing of stopping medication; prognosis, particularly in relation to subjects with a bad renal prognosis who could be targeted for more aggressive treatment; and to guide choice of therapy (e.g. cyclophosphamide versus rituximab for induction of remission). A description of recent studies examining pathogenesis and/or revealing novel genes and biomarkers of PSV that are beginning to address some of these important areas, or which provide a better understanding of biomarkers already in clinical use, is provided. It should be noted, however, that many of the studies cited (particularly in relation to AAV) deal with studies largely involving adult patients, and this important caveat must be considered when extrapolating the findings to paediatric patients.

## ANCA-associated vasculitides

The AAVs include: granulomatosis with polyangiitis (GPA), most commonly associated with proteinase 3 (PR3)-ANCA, although myeloperoxidase (MPO)-ANCAs are present in a minority; microscopic polyangiitis (MPA), typically associated with MPO-ANCA; single organ disease, including renal-limited vasculitis (again mainly associated with MPO-ANCA); and eosinophilic granulomatous polyangiitis (EGPA) [5]. As most children with EGPA are in fact ANCA-negative [5, 6], EGPA is not considered further in this review, and the reader is referred elsewhere for a recent description of this entity in children [6].

### Clinical features of AAV

From a clinical perspective, it may be useful to think of GPA as having two forms: a predominantly granulomatous form with mainly localised disease with a chronic course; and a florid, “true” acute small vessel vasculitic form characterised by severe pulmonary haemorrhage and/or rapidly progressive vasculitis or other severe vasculitic manifestation [7]. These two broad pathogenic processes may co-exist or present sequentially in individual paediatric/adult patients alike. In a series of 17 children with GPA, the frequency of different system involvement was: respiratory 87%, renal 53%, ENT 35%, musculoskeletal 53%, eyes 53%, nervous system 12%, and skin 53% [8]. Another paediatric series of GPA reported an even higher frequency of renal involvement, with 22 out of 25 patients who had glomerulonephritis at first presentation, and only 1 out of 11 patients who had renal impairment in that series recovered renal function with therapy [9]. Cabral et al. described 65 children with GPA reporting renal involvement in up to 75.4% of cases [10]. Dialysis was necessary in 7 patients (10.8%), and end-stage renal disease was present in a single patient of that series [10]. In adult patients, data suggest that approximately 73.5% of adults with GPA might have histological evidence of glomerulonephritis [11]. Of note, renal involvement in GPA accrues with increasing disease duration, which could account in part for the variation in reported renal involvement in paediatric GPA. Similar distribution of symptoms and organ involvement was noted in the cases submitted for the validation exercise of the European League Against Rheumatism/Paediatric Rheumatology International Trials Organisation/Paediatric Rheumatology European Society (EULAR/PRES/PRINTO) paediatric vasculitis classification criteria [12]. Comparing the paediatric and adult cohorts, although organ involvement, signs and symptoms are similar, there are differences in their frequencies at disease presentation; in general, adult patients have lower frequencies of constitutional symptoms (fever, weight loss), certain ENT features (oral/nasal ulceration, chronic or recurrent otitis media/aural discharge), respiratory (tracheal/endobronchial stenosis, obstruction, haemoptysis/alveolar haemorrhage), and renal (haematuria or red blood cell casts) involvement; and a higher frequency of conductive hearing loss than children [11, 13].

The typical clinical manifestations of MPA are rapidly progressive glomerulonephritis and alveolar haemorrhage, although virtually any organ system can be affected [7, 14]. In adults, 75–80% of patients have detectable peri-nuclear (p)ANCA/MPO-ANCA. MPO-ANCA pathogenicity has been established in animal models, and trans-placental transmission to a neonate has been described (see below). Renal limited forms of MPA are described in children and adults [7]. Despite the classic description that MPA mainly affects the kidneys and lungs, other organ involvement can occur and clinicians should be alert to this (7;14).

### Pathogenesis of AAV: how do ANCAs cause vasculitis?

A considerable body of evidence supports the concept that ANCAs are directly involved in the pathogenesis of small vessel vasculitis [15]. The most compelling human evidence relates to reports of a neonate who developed pulmonary renal syndrome soon after birth from the trans-placental transfer of IgG MPO-ANCA from the mother, who had active microscopic polyangiitis (MPA) [16, 17]. Moreover, mouse models show that the passive transfer of MPO-ANCA or anti-MPO lymphocytes (that make MPO-ANCA) into mice that lack functioning B or T cells (RAG −/−) resulted in pauci-immune crescentic nephritis and small vessel vasculitis similar to that observed in humans with AAV [18]. Evidence of direct pathogenicity of human PR3-ANCA had hitherto been lacking. Progress in the study of these antibodies in rodents was hampered by lack of PR3 expression on murine neutrophils, and by different Fc-receptor affinities for IgG across species. Thus, Little et al. tested whether human anti-PR3 antibodies could induce vasculitis in mice with a human immune system [19]. Chimeric mice were treated with human IgG from patients with: anti-PR3 positive renal and lung vasculitis; patients with non-vasculitic renal disease; or healthy controls. Six-days later, 39% of anti-PR3-treated mice had clinical evidence of vasculitis, which was confirmed histologically. Specifically, anti-PR3-treated mice had evidence of pauci-immune proliferative glomerulonephritis, with infiltration of human and mouse leucocytes [19], providing evidence for the first time that human IgG from patients with anti-PR3 autoantibodies was pathogenic.

A plethora of publications describe in detail the mechanism of vascular injury mediated by ANCA, and these have been eloquently summarised previously [15, 20, 21]. The most accepted model of pathogenesis proposes that ANCAs activate cytokine-primed neutrophils, leading to bystander damage of endothelial cells and an escalation of inflammation with recruitment of mononuclear cells [15, 20, 21]. Neutrophil priming involves stimulation with an agent, such as lipopolysaccharide (LPS) or the cytokine tumour necrosis factor alpha (TNF-α), at a concentration that does not of itself cause full functional activation, but rather results in the translocation of ANCA-antigen (MPO and/or PR3) from intracellular granules to the cell surface [22]. Subsequent ANCA activation of these primed neutrophils leads to a respiratory burst and degranulation, thereby generating the potential for endothelial injury. The binding of ANCAs to antigens expressed on the surface of primed neutrophils is not enough in itself to cause full neutrophil activation; the binding of Fc receptors is also necessary [23]. Following neutrophil stimulation by ANCAs, numerous cytotoxic mediators are released, including reactive oxygen species, chemokines, cytokines, proteolytic enzymes and nitric oxide (NO) [15, 20, 21]. The firm adhesion of activated neutrophils to endothelial cells results in endothelial cell damage and perhaps recruitment of other inflammatory cells including monocytes and T cells [15, 20, 21]. Following neutrophil activation by ANCA, neutrophils are driven down an accelerated, but aberrant apoptotic pathway. Thus, the neutrophils develop the morphological features of apoptosis, but there is a failure in cell surface changes, such as bi-lipid cellular membrane phosphatidylserine (PS) externalisation, which is normally an early feature of all apoptotic cells. This lack of PS externalisation means that phagocytes are less able to process the apoptotic neutrophils in a non-phlogistic fashion, explaining the well-characterised finding of leucocytoclasis that is often seen in the vasculitic lesions of certain vasculitic syndromes [24].

Thus, it is clear that ANCAs are directly pathogenic, but why do patients make ANCAs in the first instance?

### Genetic predisposition determines ANCA serotype but not clinical phenotype

Although it had long been proposed that patients with AAV had a genetic predisposition to developing the disease, convincing data in support of that were lacking [24]. In recent years, the genetic factors predisposing to ANCA development are better understood. The first ever genome-wide association study (GWAS) involved 1,233 patients from the UK with AAV; 5,884 controls; and a replication cohort of 1,454 AAV cases from Northern Europe, and 1,666 controls [25]. The strongest genetic associations were with the antigenic specificity of ANCAs, not with the clinical subtype of AAV. PR3-ANCA was associated with HLA-DP and the genes encoding alpha(1)-antitrypsin (*SERPINA1*, the main inhibitor of PR3), and the gene encoding PR3 itself (*PRTN3*). In contrast, MPO-ANCA was associated with HLA-DQ. This study thus confirmed that the pathogenesis of ANCA-associated vasculitis has a genetic component, shows genetic distinctions between GPA and MPA in terms of the ANCA serotype, but not the phenotype of AAV per se [25]. Other genetic polymorphisms, such as *CTLA4* and *PTPN22* (and probably many others) are also likely to be contributory [26]. Even though these studies provide insight into the pathogenesis of the breakdown of immune tolerance in patients with AAV, the strength of these associations neither allows these genetic polymorphisms to be used to identify high-risk populations that could be targeted for screening, nor can they be used for the diagnosis of vasculitis, to determine prognosis, or choice of therapy.

### Gene expression profiles may identify patients at risk of relapsing AAV

Even though the aforementioned single nucleotide polymorphisms detected using GWAS have little clinical utility as biomarkers, gene expression profiling may identify patients with AAV at risk of relapsing disease. McKinney et al. demonstrated that transcriptional profiling of purified CD8 T cells identified two distinct subject patient subgroups predicting long-term prognosis in AAV, and systemic lupus erythematosus (SLE) [27]. The subset of genes defining the poor prognostic group was enriched for genes involved in the interleukin-7 receptor (IL-7R) pathway, T cell receptor (TCR) signalling, and those expressed by memory T cells [27]. These subgroups were also found in the normal (healthy) population, and could be identified by measuring the expression of only three genes: *ITGA2*, *NOTCH1*, and (the aforementioned gene) *PTPN22*. Although the authors concluded that their findings could raise the prospect of individualised therapy for these high-risk patients, this approach has not yet filtered through neither to routine clinical practice, nor as part of any published clinical trial to date.

In a subsequent publication, the same group demonstrated that expression signatures of T-cell co-stimulation/exhaustion relate to outcomes in AAV [28]. Thus, they proposed that T-cell exhaustion plays a central role in determining outcome in autoimmune disease and targeted manipulation of this process could lead to new therapeutic opportunities [28].

### ANCA as a diagnostic biomarker

Although occasional cases of “ANCA-negative” AAV are acknowledged, such patients represent the vast minority and are encountered less commonly with improvements in ANCA testing methodology (including using both indirect immunofluorescence and enzyme-linked immunosorbent assay for screening), such that in modern practice most clinicians would be reluctant to make a firm diagnosis of AAV in the absence of detectable ANCA. Moreover, it is increasingly recognised that such “ANCA-negative” AAV patients have a broad differential diagnosis that may include entities such as chronic granulomatous disease, IgG4 disease, monogenic granulomatous diseases including Blau syndrome, or other immune-mediated renal pathology. That said, there are anecdotal reports of paediatric patients with vasculitis (particularly single organ disease) who were initially ANCA negative, and who subsequently became ANCA positive, suggesting that the negative predictive value of ANCA testing for patients with single organ disease at first presentation might be suboptimal [29]. Thus, the diagnostic utility of ANCA as a biomarker for AAV is largely irrefutable in the right clinical context, and when positive are especially useful for those with atypical presentations and/or single organ disease [30]. The fact that ANCA positivity is usually a requirement for enrolment into clinical trials involving patients with AAV [31], and is included in paediatric classification criteria [32] and adult definitions of AAV [33], may amplify the notion that a decreasing number of patients are ANCA negative, however.

### ANCA as a biomarker of disease activity

Irrespective of their diagnostic utility, whether or not ANCAs are useful to monitor disease activity has been long-debated [34], and an area of controversy ever since ANCAs were first discovered in 1982 [35]. In a study of 156 adult patients with GPA, decreases in PR3-ANCA level were not associated with a shorter time to remission, and increases were not associated with relapse [36]. These observations led the authors to conclude that ANCA levels cannot be used to guide immunosuppressive therapy in patients with GPA [36]. Two more recent studies agree with this conclusion overall, but suggested that the serial testing of ANCAs might have specific utility for the prediction of renal and/or pulmonary small vessel vasculitis relapse, but not for granulomatous disease. Kemna et al. studied 166 adult patients with AAV, positive for either PR3-ANCA or MPO-ANCA [37]. In this cohort, 104 patients had renal involvement. After a follow-up of 49 (±33) months, they observed that ANCA rises correlated with relapses in patients who presented with renal involvement (hazard ratio [HR], 11.09; 95% confidence interval [95% CI], 5.01 to 24.55), but in comparison, was only weakly associated with relapses in patients who presented with non-renal disease (HR, 2.79; 95% CI, 1.30 to 5.98) [37]. The authors concluded that longitudinal ANCA measurements may be useful in patients with renal involvement, but less valuable in patients with non-renal disease [37]. Even more recently, using data generated from the Rituximab Versus Cyclophosphamide for ANCA-associated Vasculitis (RAVE) trial, Fussner et al. evaluated the association of an increase in PR3-ANCA with subsequent relapse [38]. Although the authors found that an increase in the PR3-ANCA titre was not a very sensitive or specific predictor of subsequent relapse in general, they did observe some association between an increase in PR3-ANCA level and risk of subsequent relapse that was dependent on specific factors. These were:

1. PR3-ANCA detection methodology (stronger association for direct capture ELISA [hazard ratio 4.57], compared with indirect ELISA)
2. Disease phenotype (renal vasculitis [HR 7.94], or alveolar haemorrhage [HR 24.19], but not in those with a granulomatous phenotype)
3. Choice of remission induction treatment (stronger association for patients treated with rituximab as opposed to cyclophosphamide/azathioprine) [38]

These findings support the view that PR3-ANCA predominantly affects capillaritis rather than granulomatous inflammation, as highlighted in the accompanying editorial to that study [34].

### ANCA as a biomarker for choice of treatment

In another subsidiary study of the adult RAVE trial, Unizony et al. evaluated whether the classification of adults with AAV according to ANCA serotype predicted treatment response [39]. PR3-AAV patients treated with rituximab achieved remission at 6 months more frequently than patients treated with cyclophosphamide followed by azathioprine (65 vs 48%; *p* = 0.04; odds ratio [OR] 2.11, 95% CI 1.04 to 4.30). Furthermore, PR3-AAV patients with relapsing disease achieved remission more often following rituximab treatment at 6 months (OR 3.57; 95% CI 1.43 to 8.93), 12 months (OR 4.32; 95% CI 1.53 to 12.15) and 18 months (OR 3.06; 95% CI 1.05 to 8.97). There was no association between treatment and remission rates in patients with MPO-AAV, or in groups divided according to AAV diagnosis (i.e. GPA vs MPA) [39]. This study suggested, therefore, that adult patients with PR3-AAV respond better to rituximab than to conventional induction/remission maintenance treatment with cyclophosphamide and azathioprine, and that ANCA serotype may guide the type of treatment in AAV. Whether these findings can be extrapolated to paediatric patients is uncertain, although this may be explored when the results of the paediatric polyangiitis rituximab study (PEPRS) are available (https://clinicaltrials.gov/ct2/show/NCT01750697).

### Biomarkers derived from studies of neutrophil activation in AAV

A study by Hong et al. revealed insights into the pathogenesis of AAV by studying ANCA–neutrophil interactions [22]. They observed that neutrophils stimulated either with polyclonal ANCA isolated from patients with AAV or chimeric PR3-ANCA induced release of neutrophil microparticles (NMPs). These NMPs expressed a variety of markers, including the ANCA autoantigens PR3 and MPO. They bound endothelial cells via a CD18-mediated mechanism; induced an increase in endothelial intracellular adhesion molecule 1 (ICAM-1) expression and endothelial reactive oxygen species (ROS); and stimulated the release of endothelial IL-6 and IL-8. Removal of these NMPs by filtration abolished all these aspects of endothelial activation. NMPs generated from ANCA stimulation of primed neutrophils were also able to generate thrombin. Last, more NMPs were detected in the plasma of children with AAV compared with healthy controls or those with inactive vasculitis. Taken together, these observations support a role for NMP in the pathogenesis of ANCA vasculitis and could potentially suggest a target for therapeutic plasma exchange in AAV [22], even though currently, their clinical utility remains unvalidated.

Another novel aspect of neutrophil activation of relevance to the pathogenesis of AAV (although not specific to AAV) are neutrophil extracellular traps (NETs). These are chromatin fibres released by ANCA-stimulated neutrophils, containing antigenic components that include PR3 and MPO [40]. Kessenbrock et al. observed deposition of NETs in inflamed kidneys, and suggested that NET formation might trigger vasculitis and promote the autoimmune response against neutrophil components in individuals with AAV [40]. It is increasingly apparent that NETs may also be involved in various other autoimmune diseases, including systemic lupus erythematosus (SLE), and thus may offer insight into the pathogenesis of autoimmunity more generally, and possibly even a novel therapeutic target [41]. More recently, however, Wang et al. studied the potential of circulating NETs as biomarkers of disease activity in adult patients with AAV (34 with active disease, 62 in remission), and suggested that circulating levels of NETS were not useful for assessing disease activity in AAV [42]. Although serum levels of NETs in active AAV patients were higher than in healthy controls, there was no difference in circulating NETs between patients with active vasculitis and those in remission [42]. Differences in methodology for NET detection may account in part for these seemingly conflicting observations. Thus, even though NETs may provide insight into the pathogenesis of AAV, their potential as biomarkers of disease activity in AAV has not yet been realised.

### Urinary biomarkers of disease activity in AAV

Urinary biomarkers could provide an easily obtainable and non-invasive means of monitoring renal vasculitis and other renal pathology for children and adults, which, if reliable, may obviate the need for repeat renal biopsy (the gold standard) in patients suspected of having a renal flare of vasculitis [43]. Based on the observation that macrophage infiltration is important in the pathogenesis of glomerulonephritis in AAV, Tam et al. investigated urinary monocyte chemoattractant protein 1 (MCP-1), and fractalkine as potential non-invasive biomarkers for renal vasculitis in adults with AAV [44]. They observed higher urinary MCP-1 levels in patients with active renal vasculitis compared with healthy controls, patients with inactive vasculitis, and patients with extra-renal vasculitis only. There were no differences in serum MCP-1 between these patient groups and no differences in urinary fractalkine levels. Reduction in urinary MCP-1 levels following treatment preceded the improvement of renal function by a median of 2 weeks; and in one patient, rising urinary levels of MCP-1 were associated with progression to severe renal failure despite immunosuppression. The authors concluded that urinary MCP-1 could offer a useful non-invasive biomarker for the assessment of renal vasculitis, and for monitoring its treatment [44].

Recently, O’Reilly et al. suggested that urinary soluble CD163 (sCD163) may provide an even better biomarker of renal vasculitis than urinary MCP-1 [45]. They performed a thorough and in-depth study involving a rat model, adult patients with small vessel vasculitis (SVV: i.e. GPA, MPA, EGPA, and anti-glomerular basement membrane disease), healthy controls, and disease controls. Disease controls were subdivided into those with and those without glomerulonephritis, and intensive care patients with non-immune-mediated acute kidney injury, multisystem failure and sepsis. They detected sCD163 in rat urine early in the disease course of experimental vasculitis. Furthermore, micro-dissected glomeruli from patients with SVV had higher levels of CD163 mRNA expression than those with lupus nephritis, diabetic nephropathy, or nephrotic syndrome, and there was strong expression of CD163 protein in the glomeruli and interstitium of patients with SVV. Patients with SVV had markedly higher urinary sCD163 than patients in remission, disease controls, or healthy controls. These results were confirmed using both internal and external validation cohorts. The authors suggest that an optimal cut-off for urinary sCD163 0.3 ng/mmol creatinine would detect active renal vasculitis with a sensitivity of 83%, specificity of 96%, and positive likelihood ratio of 20.8. They concluded that this urinary biomarker could be useful for monitoring disease activity in renal vasculitis in patients with an already established diagnosis, but not necessarily for the diagnosis of renal vasculitis in the first instance, as some of the sepsis patients had high levels. They also highlighted that urinary sCD163 is a highly stable protein in urine (stable for at least a week at room temperature) and was better suited to storage/transport than urinary MCP-1, which degrades quickly [45]. Future studies should attempt to replicate these findings in paediatric patients before this test can be used reliably in children with renal vasculitis, however.

## Other biomarkers and miscellaneous vasculitides: endothelial injury and repair concept

Systemic vasculitis is characterised by systemic inflammation, endothelial activation, and (in some cases) necrosis of blood vessel walls, leading to multi-organ injury [24, 46]. Endothelial activation and injury are central to the pathogenesis, with increased endothelial cell adhesion molecule expression, and a switch to a prothrombotic endothelial phenotype, both of which contribute to the vascular pathology of vasculitis [47, 48].

Two important biomarkers of endothelial injury are endothelial microparticles (EMPs), and circulating detached mature endothelial cells (CECs). EMPs are released from the cell surface in response to a variety of stimuli associated with endothelial injury, including vasculitis and atherosclerosis [49]. It has previously been demonstrated that endothelial injury in vasculitis can be tracked through the detection of circulating EMPs [49–52]. More recently, studies have revealed that these EMPs may generate excess thrombin and thrombotic complications in children with systemic vasculitis [53], cerebral vasculitis of the young [54], and as mentioned above, MPs may be an important target for therapeutic plasma exchange in AAV [22]. CECs are necrotic, highly activated endothelial cells that detached from the vessel wall [55]. They have been described as a biomarker of disease activity in vasculitis in adults [55] and children [50, 51], childhood stroke caused by cerebral vasculitis [54], as a marker of coronary injury years after Kawasaki disease (KD) [56], and many other clinical scenarios associated with endothelial injury.

In addition to this severe endothelial injury, it has been suggested that endothelial repair processes may also be impaired in certain clinical scenarios [57, 58], and thus vasculitis could be particularly damaging to the cardiovascular system owing to an unfavourable imbalance between endothelial injury and repair, as is the case in other diseases targeting the endothelium [59, 60]. It is now known that recruitment of bone marrow-derived endothelial progenitor cells (EPCs) represent an important mechanism of endothelial repair [61]. These EPCs may play an important role in endothelial maintenance and vascular healing in health and disease, and are emerging as important biomarkers of prognosis in atherosclerosis [62, 63]. As vascular dysfunction is an important late sequela of vasculitis in children [64] and adults [65], recent studies have focussed on the perturbation of endothelial repair pathways. Zavada et al. observed a reduced number of endothelial progenitor cells in adults with AAV, and found that they were predictive of early relapse [66]. Intriguingly, and in contrast to this finding in adults, Clarke et al. observed numerically higher circulating levels of EPCs in children with active vasculitis, suggesting that paediatric patients might be able to mount a compensatory repair response to inflammatory endothelial injury [51]. Hong et al. observed, however, that the function of these elevated circulating EPCs was impaired in children with active vasculitis [47]. Similar findings were observed by Eleftheriou et al. in children with arterial stroke caused by cerebral vasculitis [67].

Von Willebrand factor (VWF), a plasma protein synthesised primarily by megakaryocytes and endothelial cells, mediates platelet aggregation and adhesion [68]. Levels increase in response to endothelial injury or activation, and have been described as a biomarker of disease activity in many vasculitides including KD, HSP and AAV. Its performance as a biomarker of disease activity in childhood CNS vasculitis was recently investigated. In a cohort study of 39 children with childhood primary angiitis of the CNS, 65% had increased levels at diagnosis, which decreased significantly after treatment. The authors concluded that although these preliminary results were encouraging, further studies are required to evaluate the sensitivity and specificity of VWF as a potential biomarker for disease activity in this context [68]. Indeed, another study of children with cerebral vasculitis found VWF to be a poor discriminator of disease activity [54]. More recently, a study of VWF in adult patients with AAV and renal involvement observed high levels of VWF in patients with active vasculitis that persisted even when they were considered in clinical remission [69]. This casts some doubt on the validity of VWF as a robust biomarker of disease activity in AAV. Moreover, whether elevation of VWF solely represents endothelial activation or injury, or alternatively platelet activation and/or activation of the clotting cascade, is debatable [69].

Thus, the endothelial injury and repair concept provides a framework for studies that provide insight into the pathogenesis of systemic vasculitis and its sequelae. Even though in the future this may provide useful biomarkers for monitoring disease activity, response to treatment, defining prognosis, or identify new therapeutic targets, these biomarkers (with the exception of VWF) remain (at the moment) within the research domain.

## IgA vasculitis (Henoch–Schönlein purpura)

IgAV is one of the most common vasculitides of childhood [70], and is of particular relevance to the paediatric nephrology community. The clinical picture is characterised by non-thrombocytopaenic purpura, arthritis and arthralgia, abdominal pain, gastrointestinal haemorrhage, and glomerulonephritis. The American College of Rheumatology (ACR) classification criteria [71] are now superseded by paediatric classification criteria [32]. There has been a lack of progress in relation to understanding the pathogenesis of IgA vasculitis, and therapeutic clinical trials, particularly for the associated glomerulonephritis. It is hoped that robust paediatric classification criteria will facilitate future high-quality translatable research to make progress in this area.

### Pathogenesis of IgAV: genetic background

Several polymorphisms relating to disease susceptibility, severity, and/or risk of renal involvement have been described. Studies of this nature have been hampered by relatively small patient numbers and thus lack the power to be definitive or necessarily applicable to all racial groups. It is, however, increasingly apparent that the genetic contribution is complex and probably polygenic in nature, although familial clusters of the disease may occur. Investigations from Spain have presented preliminary data on genetic associations. The frequency of HLA-B35 was increased in patients who developed nephritis [72]. The incidence of HLA-DRB1∗01 was also increased compared with matched controls, and HLA-DRB1∗07 was decreased [73]. A study of unselected children with IgAV from Turkey showed that HLA A2, A11 and B35 antigens were associated with a significantly increased risk of IgAV, whereas HLA A1, B49 and B50 antigens were associated with a decreased risk of the disease [74]. There was no association, however, with HLA class 1 alleles and renal involvement [74]. Even though there were no general associations with the expression of intercellular adhesion molecule 1 in patients compared with controls, a K/E polymorphism at codon 469 was significantly decreased in those who did not develop severe gastrointestinal manifestations (and possibly in patients without renal sequelae) [75]. In studies from Israel and Turkey, mutations in the familial Mediterranean fever (MEFV) gene were frequent in patients with IgAV [76, 77]. Other genetic polymorphisms have also been implicated, and have been reviewed elsewhere [78].

### Immunology of IgAV and potential biomarkers

Many reports have implicated infection, particularly with β-haemolytic streptococci, as a potential trigger for this disease [79–82]. Some investigators have, however, doubted this association [83–85]. Other preceding immunological triggers, including vaccination [86, 87] and viral infection [88–91], have been described.

The characteristic vascular deposition of IgA strongly suggests that IgAV might be a predominantly IgA-mediated dysregulated immune response to antigen and may operate through the alternative complement pathway [92]. Although the pathogenetic mechanisms of nephritis are still not delineated, studies suggest that galactose-deficient IgA1 might be recognised by antiglycan antibodies, leading to the formation of circulating immune complexes and their mesangial deposition, which results in renal injury in IgAV [93]. An increase in the levels of poorly galactosylated IgA1 appears insufficient to cause IgAV nephritis or IgA nephropathy, as first-degree relatives may have high serum levels of poorly galactosylated IgA1, but no signs of nephritis [94, 95]. Thus, it is likely that a “second hit” is required for high levels of poorly galactosylated IgA1 to form immune complexes that result in nephritis [95]. It is suggested that this second hit might be the formation of antiglycan IgG or IgA antibodies (perhaps triggered by infection), that then go on to form large circulating immune complexes with the poorly galactosylated IgA1, which are prone to deposition [95]. Currently, the genes controlling IgA1 glycosylation are unknown. Other factors that may modulate IgA synthesis and galactosylation include B-cell programming at the time of antigen encounter, Toll-like receptor activation, and local cytokine production [95]. It has been suggested that the genetic mechanisms controlling these processes should be further explored to try to better understand the genetic contribution to the pathogenesis of HSP nephritis, and IgA nephropathy [95]. Thus, even though many questions remain unanswered in relation to this pathogenetic model, increased serum levels of poorly galactosylated IgA1 remain the most consistent finding in patients with IgAV nephritis and IgA nephropathy, and these almost certainly predispose to the formation of IgA immune complexes with resulting vasculitis.

Disorders of coagulation and its activation are also associated with the development of IgAV [96]. It is recognised that disease activity may be linked to a rapid decline of factor XIII, particularly in patients with severe abdominal involvement [97–99]. This may be useful as a prognostic or diagnostic biomarker because the decline occurs before classic skin rash and thus could allow early diagnosis of IgAV, where abdominal symptoms and signs predominate. There is no information regarding the diagnostic specificity of a fall in factor XIII, however. Anecdotal reports of factor XIII replacement to successfully treat severe abdominal symptoms in IgAV are described [99], but no high-quality controlled data relating to this treatment are available. Factor XIII also declines before recurrence of IgAV; yet, it is unknown whether this is merely a secondary epiphenomenon, or a true causative association.

Makay et al. recently suggested that an elevated circulating neutrophil to lymphocyte ratio (NLR) might be a predictor of gastrointestinal haemorrhage in children with IgAV [100]. This finding was not replicated in a separate study that excluded those already bleeding, which is of particular relevance as acute haemorrhage is associated with neutrophilia [101]. Thus, the clinical utility of the NLR in IgAV is questionable.

## Monogenic vasculitides: DADA2, CANDLE and SAVI

Three recently described monogenic autoinflammatory conditions with a major vasculitic component are worthy of a brief description, as they highlight new pathophysiological pathways with potential for targeted treatment, provide new biomarkers and serve to emphasise the concept that understanding the molecular pathogenesis may lead to more targeted therapy [102–105].

### DADA2

Deficiency of adenosine deaminase type 2 (DADA2) is an autosomal recessive disease resembling polyarteritis nodosa, caused by homozygous or compound heterozygous mutations in the *CECR1* gene [102, 103]. The cardinal clinical features include livedo racemosa, neurological involvement including the propensity to lacunar (small vessel) stroke, vasculitic peripheral neuropathy, digital ischaemia and cutaneous ulceration, systemic inflammation, and other end organ damage [102, 103, 106, 107]. There is an emerging view that anti-TNF-alpha therapy is particularly efficacious for this form of monogenic vasculitis [107]; this may be because the extracellular enzyme ADA2 functions as an important regulator of immune development. Patients with DADA2 demonstrate skewed macrophage development towards the M1 pro-inflammatory phenotype as opposed to the M2 anti-inflammatory phenotype [102, 103]. M1 macrophages are known to produce TNF-α, which could explain why this therapeutic approach seems particularly effective in DADA2 [102, 103]. Allogeneic haematopoietic stem cell transplantation has been reported to be successful in a few patients [108]; gene therapy may be an option for the future [107].

In relation to a novel diagnostic biomarker for this form of vasculitis, an obvious candidate would be serum or plasma adenosine deaminase type 2 activity and/or levels. These assays are beginning to be established in some centres, although mainly within the research domain at the moment. Thus far, preliminary data indicate that healthy children have higher ADA2 activity levels than adults, a fact that should be taken into account when considering using this test as a diagnostic biomarker for DADA2 [109]. In addition, ADA2 enzyme activity appears not to change with disease activity, as pre-symptomatic patients with confirmed genetic mutations in *CECR1* have low levels similar to those in patients with active disease and patients in remission on treatment [109].

Even if molecular genetic testing becomes more widely available for this disease, it will still be important to have confirmatory ADA2 enzyme activity or a level assay to understand whether or not any novel genetic sequence variants (“variants of unknown significance”) in *CECR1* are truly pathogenic. Thus, genetic screening for DADA2 should be backed up with some assessment of ADA2 enzyme activity and/or level before a firm clinical diagnosis can be established. Validated methodologies with international and age-specific standards will be important to establish full incorporation of this biomarker into routine clinical care for children.

### CANDLE and SAVI

Chronic atypical neutrophilic dermatosis with lipodystrophy and elevated temperature (CANDLE) syndrome is a recessive disease caused by homozygous, compound heterozygous or digenic mutations in the proteasome pathway, and is classified as a proteasome-associated autoinflammatory syndrome (PRAAS) [105, 110]. Mutations in *PSMB8*, *PSMB4*, *PSMB9*, *PSMA3*, and proteasome maturation protein (*POMP*) are described [105, 110]. In the early stages, the neutrophilic dermatosis may display the histological features of neutrophilic/leucocytoclastic vasculitis. CANDLE is associated with dysregulated type I interferon production; therefore, targeting this pathway with Janus kinase (JAK) inhibitors may be a promising novel therapeutic approach [105, 110]. Early clinical trials of baricitinib, an oral JAK1 and JAK2 inhibitor, are ongoing.

Stimulator of interferon genes (STING)-associated vasculitis of infancy (SAVI) arises from sporadic/dominant mutation in the *TMEM173* gene and presents early in life with a vasculitic rash affecting the cheeks, nose and peripheries with chronic ulceration; and progressive interstitial pulmonary fibrosis and associated pulmonary hypertension [104, 111]. Standard vasculitis therapies are ineffective. Cutaneous vasculitis and deteriorating lung function usually continue relentlessly throughout childhood, with development of pulmonary hypertension and lung fibrosis, often with fatal outcome [104, 111]. SAVI is considered part of the growing group of Mendelian disorders recognised as “interferonopathies,” which include Aicardi–Goutières syndrome (AGS), and the aforementioned CANDLE syndrome [112]. Although virtually no published data are currently available, anecdotal reports again suggest that early treatment targeting the interferon pathway (e.g. with JAK inhibitors) might currently offer the best hope for survival.

Gene expression profiles (an “interferon score”) can be used to screen for suspected interferonopathies, and thus can be exploited as diagnostic screening biomarkers to guide genetic screening and/or interpretation [113]. Interferon gene expression profiles could also be used as a biomarker to guide choice of therapy (e.g. JAK inhibition versus more conventional vasculitis treatment), and to monitor disease activity in response to therapy [113]. Again, a few centres are beginning to establish this approach, but at the moment this is mainly still being researched and is not yet widely available.

## Conclusion

Considerable progress has been made in understanding the pathogenesis of vasculitis, and consequently promising biomarker discoveries. Despite this, it should be remembered that primary outcome measures in clinical trials of vasculitis remain clinical, such as the paediatric vasculitis activity score [114] and its adult equivalent. In particular, novel diagnostic tests, particularly for monogenic forms of vasculitis, which can be screened for using next-generation sequencing targeted gene panels, are highlighted, as these particular monogenic forms of vasculitis have significant therapeutic implications, and demonstrate that an understanding of the molecular basis of vasculitis can identify new therapeutic pathways, new biomarkers and new treatments.
